# Using the Veggie Meter in Elementary Schools to Objectively Measure Fruit and Vegetable Intake: A Pilot Study

**DOI:** 10.3390/mps4020033

**Published:** 2021-05-12

**Authors:** Sarah Martinelli, Francesco Acciai, Natasha Tasevska, Punam Ohri-Vachaspati

**Affiliations:** Nutrition Program, College of Health Solutions, Arizona State University, Phoenix, AZ 85004, USA; facciai@asu.edu (F.A.); natasha.tasevska@asu.edu (N.T.); punam.ohri-vachaspati@asu.edu (P.O.-V.)

**Keywords:** fruit and vegetable consumption, elementary school children, skin carotenoids, diet assessment, Veggie Meter©

## Abstract

Self-reported fruit and vegetable (FV) consumption in children has limitations that could be overcome with objective measures that are easy to implement. The Veggie Meter (VM) is a non-invasive portable device that measures skin carotenoid levels, a concentration biomarker of usual FV intake. While VM has been used to measure FV consumption in adults, few studies have explored its use in elementary school settings. Designing research studies using the VM with elementary school-age children requires an understanding of how well this device can be used in a school setting and of the distribution of VM scores in this population. We used VM to measure skin carotenoids in a diverse sample of 143 elementary school children who also answered commonly asked questions about consumption frequency of FV the previous day. Multivariable regression was used to assess the independent association of demographic variables with VM scores. VM scores were also compared with student-reported FV intake. There was a weak but statistically significant correlation between reported frequency of total vegetable consumption the previous day and observed VM scores (r = 0.174, *p* = 0.042). This study provides an example of the successful use of the VM in a school setting to collect an objective measure of FV intake and provides important description of data that can inform future studies.

## 1. Introduction

Adequate fruit and vegetable (FV) consumption is crucial for overall health [1], yet consumption remains low, especially in children [2,3,4]. Interventions aimed at increasing FV consumption in children are widespread in school and community settings [5,6]. Traditional methods of assessing consumption based on self-reports are prone to random and systematic measurement errors and are challenging to implement in younger children [7,8,9,10]. Therefore, other approaches, based on biomarkers as measures of intake, have been developed. For instance, the concentration of carotenoids in the serum is considered the best available biomarker for assessing FV intake, as FV are high in carotenoids and carotenoids cannot be synthesized by the body [11]. The downside of this method is that it requires collecting a blood sample; thus, it is invasive, difficult to implement in children, and can be costly. To overcome these issues, non-invasive optical technologies for objective measurement of carotenoid levels in the skin, including resonance Raman spectroscopy (RRS) and pressure-mediated reflection spectroscopy (RS) have recently emerged [12]. These technologies rely on the unique light-scattering properties of carotenoids and their storage in the upper layers of the skin. Dietary carotenoids are deposited in the skin via lipid transporters and are absorbed into the skin when secreted with sweat onto the skin [13].

The RRS method has been validated against skin biopsies in adults [14] and against serum carotenoid levels in both adults [15,16,17] and children [18,19]; in addition, it has been shown to respond to changes in dietary intake as soon as two weeks after the start of a dietary intervention high in carotenoids [16]. However, the RRS approach is not itself without limitations, due to lack of portability and limited availability of the RRS device outside of research institutions. While the RS technology operates under similar principles as the RRS (i.e., it relies on reflection of light off the unique structure of carotenoid molecules stored in the skin), devices that use this technology are readily available for purchase, are easy to use, and are portable. The most widely used RS device is the Veggie Meter© (VM) (Longevity Link Corp., Salt Lake City, UT, USA), which is strongly correlated with plasma carotenoids—the gold standard for measurement of dietary carotenoids [17,20]—and has been validated against food frequency questionnaires [21] and RRS [17,20] in adults. To date, research using the VM with elementary aged children is limited, especially in school settings [6,22]. Two studies conducted in children, thus far, did not include elementary school age students [6,22]. To help future research, the aim of this study was to assess if the VM could be easily used in an elementary school setting. We also examined the distribution of skin carotenoids among elementary school age children and explored its variation by demographic characteristics. Lastly, we compared VM measurements with self-reported FV intake on the previous day assessed using selected questions from the School Physical Activity and Nutrition (SPAN) survey [23]. While self-reporting of FV consumption is not a gold standard, SPAN questions are frequently used to assess FV consumption in school settings.

## 2. Materials and Methods

### 2.1. Study Design

A convenience sample of school and district-level personnel from two school districts in Phoenix Arizona, one in a higher income area and one in a lower income area, were approached to identify elementary schools for participation in the study. Five school principals contacted with a written request agreed to participate. Aims of the study were shared with the principals, who were also informed that data collectors would ensure minimal disruption to classroom instruction. Given the non-invasive nature of the data collection, the low burden placed on participants, and student data were not identified or tracked over time, principals in four schools provided consent in loco parentis. In one school, parents were sent a letter describing the study and could opt their child out by returning the letter to the teacher. No parent returned the form to keep their child from participating in the study. On data collection days, two trained data collectors introduced the study to the classroom teacher and students and obtained oral assent from each student. Data from all five schools were collected in November 2019. 

### 2.2. Participants

Data were collected from 154 4th–6th graders (aged 9–11) attending the five elementary schools that consented to the study. The proportion of student eligibility for free or reduced-price meals was used as a proxy for school-level income. Two schools in the sample had a student population with free and reduced-price meal eligibility at or above 89%. Students attending these schools were categorized as attending low-income schools. The other three schools had a student population with free and reduced-price meal eligibility between 4% and 13%. Students attending these schools were categorized as attending high-income schools. Data on eligibility for free and reduced-price meals were obtained from the National Center of Education Statistics for the 2018–2019 school year, the most recent year for which data were available [24]. 

### 2.3. Measures

Classroom data collection started with students completing a subset of questions focusing on FV consumption on the previous day from the SPAN [23], a commonly used instrument for assessing FV intake in similarly aged children [25,26,27,28]. Students reported the number of times they consumed fruit (not including fruit juice) and vegetables from 5 subgroups: starchy (including potatoes that are baked but not fried), green, orange-colored, beans, and other vegetables, on the day prior. Examples of questions included: “Yesterday, did you eat orange vegetables like carrots, squash, or sweet potatoes?” Response options included “No I didn’t eat any orange vegetables yesterday”, “Yes, I ate orange vegetables 1 time yesterday”, “Yes, I ate orange vegetables 2 times yesterday”, or “Yes, I ate orange vegetables 3 or more times yesterday”. Students also reported their age, sex, race, and grade level in the survey. Survey questions were read out loud by data collectors as students provided responses on paper copies. 

After survey completion, students were invited, in groups of 1 to 2, to a measuring station set up at the back of the classroom for a VM scan. Students were asked to show the data collector which hand they write with to identify the dominant hand. The index finger of the non-dominant hand was then cleaned with an alcohol wipe to reduce any potential interference from contamination before the VM scan. To avoid confounding due to differential levels of melanin in the skin, the VM protocol uses the finger, which has lower melanin levels compared to other areas of skin, as the primary scanning site. The instrument also uses an algorithm to account and correct for melanin in the skin [29]. Three consecutive scans were taken (with a 5-s pause in between), which the VM averaged to generate a single VM score. The same VM machine was used in each classroom in the study. The machine was calibrated according to the manufacturer’s instructions prior to each data collection period. At the end of data collection students received a lanyard for participating in the study.

VM measurements were completed in each classroom within a classroom period with minimal disruption to regular instruction. Summary of de-identified VM data from each classroom was provided to the teacher with a brief explanation, who then shared the findings with the class as part of an experiential scientific project.

The study was approved by the Institutional Review Board at Arizona State University. 

### 2.4. Statistical Analysis

From a total of 154 participants, complete data on VM measurements were available for 144 students. Six students opted out of the VM scan, two students were not scanned because of time constraints, and VM scores were not recorded for two students due to finger contamination that interfered with the VM reading. After examining studentized residuals of VM scores (generated from a multivariate regression model that included student age, sex and school income), one case was identified as an outlier and was dropped. The result was a total sample of 143 students with VM scores. The distribution of VM scores was normal, based on the Shapiro–Wilk test for normality (*p* = 0.458), after the outlier was removed. Lastly, the five students who had at least one missing value on any one of the 6 questions about FV consumption from the survey were removed from the correlation analysis between VM scores and the SPAN (*n* = 138). 

Three consumption variables were generated as follows: “total vegetable” (5 vegetable subgroups: starchy, green, orange-colored, beans, and other vegetables), “total orange/green vegetable” (two vegetable subgroups: orange- and green-colored vegetable) and “total fruit”. Extreme values from three students were top coded so that reported intake was not greater than three standard deviations from the mean for any consumption frequency score. 

VM scores were divided into quartiles to compare scores in the extreme categories and mean and standard deviation of VM scores were calculated within each quartile. Two sample *t*-tests and analysis of variance (ANOVA) were used to generate descriptive statistics for VM scores across different demographic measures both with the continuous measures of VM scores and VM quartiles. Next, we examined Pearson’s correlation between VM scores and each of the consumption variables. Finally, we ran multivariable linear regression with VM score as the dependent variable and student level demographics as independent variables (sex, age, race, and income-level), while accounting for school level clustering. All statistical analyses were conducted in Stata version 15 (StataCorp, College Station, TX, USA). Tests were considered significant at *p* < 0.05.

## 3. Results

**Figure 1:**
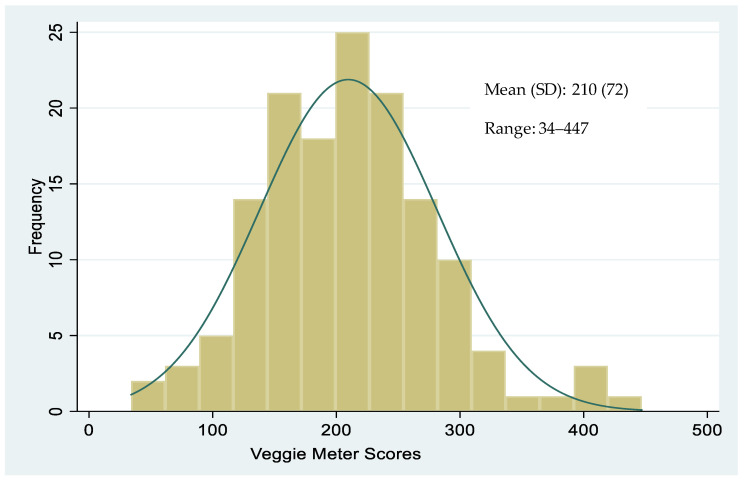
Distribution of Veggie Meter scores in a diverse sample of elementary school children (*n* = 143).

Each of the five schools contributed between 14 and 27% of the total sample (*n* = 143). VM scores ranged from 34 to 447 (the complete range of possible scores goes from zero to 800), and the mean and standard deviation of VM score for all students was 210 (±72) (Figure 1). The bivariate analyses showed that VM scores did not differ across child’s sex, age, or race (Table 1). However, students from high-income schools had lower mean VM scores (201 ± 80) compared to students from low-income schools (221 ± 59), and the difference was statistically significant (*p* = 0.044). 

Table 2 summarizes the results from the multivariate linear regression analysis examining the association between VM scores and demographic characteristics, adjusting for students being nested within schools. VM scores and income-status and child age were inversely related, indicating that students from higher-income schools and older students tended to have lower VM scores. These relationships approach significance (*p* = 0.082 and *p* = 0.088, respectively). No associations were observed for child sex or race.

There was a weak but statistically significant correlation between reported frequency of total vegetable consumption and VM scores (r = 0.174, *p* = 0.042). No significant correlations were observed between the other consumption variables and VM scores (total orange and green vegetable r = 0.113, *p* = 0.185; and total fruit r = 0.048, *p* = 0.571). To explore this relationship further, we examined the mean consumption patterns for children in the four VM score quartile-based categories (Table 3). It is interesting to note that while the highest consumption values for total vegetables were observed for children in the top VM score quartile, the mean values for consumption for the lower-three quartiles did not follow a pattern. 

## 4. Discussion

To date, no studies have used the Veggie Meter to objectively measure fruit and vegetable consumption among elementary school age children. Using data from a diverse elementary school population in five schools in Phoenix, Arizona, this study shows that it is feasible to collect VM data in school settings and describes the distribution of VM scores, overall and by student demographics. VM scores ranged between 34 and 447 and were normally distributed. There were sociodemographic differences in VM scores, students in low-income schools having higher scores than students in high-income schools, on average. VM scores tended to decrease with age, even though this relationship did not reach significance, perhaps because of the smaller sample size.

Given the known limitations of self-reported dietary recalls [9], especially in children [7,8], the use of the VM could be an important instrument for nutrition research in the school setting. In a recent study where the VM was used with pre-school students from Head Start classrooms in North Carolina, the VM mean score was 266 (±83) [22], somewhat higher than what we found (210 ± 72). Similarly, another study using the VM with preschool students (ages 3–8) reported a higher range of VM scores [6]. Lower VM scores in our study could be because our sample included older children (ages 9–11), who tend to have a lower consumption of FV compared to recommendations [30,31]. Even within our restricted age range, there is some evidence that older children tend to have lower FV intake. These results are in line with recent studies using data from the National Health and Nutrition Examination Survey (NHANES), which show that older children have an overall lower diet quality than younger children, mostly because of lower fruit and whole grain intake and higher intakes of sugar sweetened beverages [2,3,4].

In bivariate analysis, students from lower-income schools had higher VM scores compared to students from high-income schools. This result is surprising as low-income children are consistently shown to consume fewer FV than their higher-income counterparts [3,4,32]. However, higher VM scores in low-income students in this sample may be due to their participation in the National School Lunch Program (NSLP). The NSLP provides lunch to students during the school day at low to no cost and requires meals meet specific nutrition guidelines set out by the United States Department of Agriculture [33]. Participation in NSLP is higher in low-income schools where more children receive free and reduced-price meals [34]. The Healthy Hunger Free Kids Act (HHFKA), passed in 2010 and implemented starting in 2012, increased requirements for the availability and variety of FV offered as part of NSLP to better align with public health recommendations [35]. Previous studies have found that consumption of FV has increased in students participating in school meals [36,37,38,39] and that the overall diet quality of students consuming school meals is higher than students who do not consume school meals since HHFKA was enacted [40].

We found weak but significant correlation between reported total vegetable intake the day prior using a subset of the SPAN and VM scores, while no correlation was observed between the VM scores and reported total orange and green vegetable or total fruit intake. These results are consistent with those reported by May et al. [22] who also saw no correlation between VM scores and total reported FV consumption frequency measured using the SPAN in a sample of 94 middle school students (r = 0.056, *p* = 0.596). This weak or lack of correlation is not surprising. In fact, while VM is a measure of usual intake, the SPAN examines intake from only one day and is prone to measurement errors typical of self-reported consumption measures. In our sample, we did see that students who reported the most FV intake were in the highest VM score quartile; however, we did not see a trend in reported consumption across the other quartiles. SPAN may be good at separating the highest consumers from the others based on the prior days consumption but may not discriminate between other levels of consumption. The VM provides more accurate measures of usual intake than commonly used survey measures that are only capturing intake the previous day.

Carotenoid levels in the skin have been shown to mirror changes in carotenoid consumption in controlled feeding interventions [16] and after using carotenoid supplements [15,41]. While blood carotenoids and VM scores are highly correlated, blood concentrations respond to change in diet relatively faster than skin measures [15,41]. As a result, skin carotenoid measures yield a more stable measure of exposure, reflecting intake over the previous two months, compared to plasma or serum carotenoids, which have shorter half-lives [16]. When VM scores in adults were compared with serum carotenoids, the gold standard biomarker of FV intake, correlations were high (r = 0.71 and 0.81; *p* < 0.001) [20,21]. Jahns et al. also found that correlations between skin carotenoids measured using RS and blood carotenoids were moderate to strong, while 24-h recalls showed weak to no correlations with skin or blood carotenoids [17]. In a recent study, VM measures have been shown to be highly reliable for measuring FV intake changes over time within the same participant [20]. While skin carotenoids measured using RRS technology have been validated with serum carotenoid levels in children [18,19,42,43], VM measurements using RS technology have not yet been validated with serum carotenoids in children.

Even in the absence of comparisons with the established biomarker among children, based on validation studies in adults, correlation with RSS values, and given the ease of its use, the RS technology has become more commonly used. For instance, it was used in an evaluation study in a community setting with young children (3–8 years old) to assess the impact of a health education intervention aimed at increasing FV consumption, delivered to their parents through cell phones and social media platforms [6]. FV consumption was captured using photos of foods consumed, a pre- and post-survey regarding FV consumption, and VM scans. While photos of foods consumed did not appear to reflect a change in consumption, the intervention group did report higher vegetable consumption in the post survey and had higher VM scores post intervention compared to the control group [6]. Furthermore, starting in 2016, the city of San Francisco added the VM as a public health screening tool for preschool children and used the VM to teach children about the benefits FV consumption and to inform public health planning [20]. Similar to our findings in elementary school age children, a recent study in pre-school, middle, and high-school children found weak associations between VM scores and self-reported measures of FV liking (in preschoolers) or intake frequencies (measured by the SPAN in middle school students and the National Cancer Institute screener in high-school students) [22]. Interestingly, the authors reported negative and statistically significant associations between soda consumption, used as a proxy measure for overall diet quality, and VM scores in middle-school students.

This study is not without limitations. First, our study is based on a convenience sample; yet there is representation from predominant race/ethnicity groups as well as from schools with low and high rates of free and reduced-price meals. We did not have information on children’s consumption of vitamin supplements, which can impact VM scores. In the US, about a quarter of children consume vitamin supplements [44] and this practice is lower in lower-income and minority populations [45]. Further contamination of skin from colors in food can impact VM readings. While we cleaned the finger on which readings were made and we used the child’s non-dominant hand, which is less likely to be contaminated, it is possible that some pigmentation remained. In addition, carotenoid levels present in skin or blood reflect the consumption of not only FV that are high in carotenoids but also other foods, such as eggs, salmon, and shellfish, which could potentially confound results. In addition, carotenoid values can be affected by factors other than diet (e.g., genetic variation [46], sun exposure, adiposity [47]). Therefore, the use of RS technology might be best employed when tracking the same individuals over time, rather than in a cross-sectional analysis of a given population. Finally, we were not able to collect and control for student height and weight for this study. Future studies should investigate the feasibility of including student height and weight measures along with VM scans to adjust for adiposity. A major strength of this study is that it provides data on VM scores in a diverse sample of elementary-aged students, a population not yet covered in the literature. In addition, we provide VM scores by age, sex, race and income categories, information that may be useful for future studies as well as for designing interventions and evaluations.

## 5. Conclusions

This study shows the feasibility of using the VM as an objective measure of FV intake in a classroom setting. We present distribution and range of VM scores by demographic characteristics of elementary school-age children. There was limited association between VM scores, which capture usual consumption, and consumption reported for the previous day by a commonly used SPAN questionnaire. Objective measurement of FV consumption can overcome the inherent biases of self-reported measures and may improve the assessment of interventions aimed at increasing the intake of FV in school-aged children.

## Figures and Tables

**Table 1:** Mean Veggie Meter (VM) scores by demographic characteristics of the sample (*n* = 143) ^1^.

		*n*	(%)	Mean	(SD)	*p*-Value
Sex	Boys	68	(47.6)	210	(74)	0.495
	Girls	75	(52.4)	210	(70)	
Age	9 y	39	(27.3)	222	(81)	0.404
	10 y	51	(35.7)	209	(66)	
	11–12 y	53	(37.1)	201	(70)	
Race	Hispanic	65	(45.5)	216	(66)	0.326
	Non-Hispanic White	53	(37.1)	211	(79)	
	Other	25	(17.5)	191	(70)	
School Income Level ^2^	Low	63	(44.1)	221	(59)	0.044
	High	80	(55.9)	201	(80)	

^1^ Bivariate analysis. Analysis of variance tests used for examining association of VM scores with age and race and *t*-tests for association of VM scores with sex and school income level; ^2^ Based on school-level participation in free and reduced-price meals reported by NCES.

**Table 2:** Results from linear regression models examining the association between Veggie Meter scores and demographic characteristics (*n* = 143) ^1^.

		β	*p*-Value	95% CI	
Sex	Boys	Reference			
	Girls	−1.0	0.954	−48.4	46.3
Age	9 y	Reference			
	10 y	−24.6	0.128	−60.3	11.0
	11–12 y	−28.4	0.088	−63.4	6.7
Race	Hispanic	Reference			
	Non-Hispanic White	26.7	0.313	−37.5	90.9
	Other	−9.3	0.732	−79.7	61.0
School Income Level ^2^	Low	Reference			
	High	−39.7	0.082	−87.5	8.0

^1^ Analysis adjusted for the nesting of students within schools; ^2^ Based on school-level participation in free and reduced-price meals.

**Table 3:** Veggie Meter scores and reported fruit and vegetable consumption frequencies by quartile categories of Veggie Meter scores.

			VM Score Quartile Categories							
	Full Sample		Q1		Q2		Q3		Q4	
	Mean	(SD)	Mean	(SD)	Mean	(SD)	Mean	(SD)	Mean	(SD)
VM score (VM units) ^1^	210	(72.0)	125	(32.0)	184	(14.0)	228	(12.0)	304	(50.0)
Fruit and Vegetable Consumption (Frequency/day) ^2^										
Total Fruit/Vegetable ^3^	3.9	(3.2)	3.8	(3.1)	3.6	(2.9)	2.9	(2.0)	5.4	(4.0)
Total Vegetable ^3^	2.6	(2.6)	2.5	(2.5)	2.3	(2.1)	1.7	(1.6)	3.8	(3.4)
Total Orange/Green Vegetable ^4^	1.1	(1.4)	1.1	(1.5)	0.9	(1.4)	0.9	(1.2)	1.5	(1.7)
Total Fruit ^4^	1.3	(1.1)	1.3	(1.1)	1.4	(1.2)	1.2	(1.0)	1.5	(1.0)

^1^ estimates based on data from 143 elementary age students, ^2^ estimates based on data from 138 elementary age students, ^3^ *p*-value from analysis of variance (ANOVA) differences between quartiles <0.05, ^4^ *p*-value from ANOVA for difference between quartiles <0.10.

## Data Availability

The data presented in this study are available on request from the corresponding author. The data are not publicly available due to the institution’s IRB requirements.
